# Preferences for a Mental Health Support Technology Among Chinese Employees: Mixed Methods Approach

**DOI:** 10.2196/40933

**Published:** 2022-12-22

**Authors:** Sijin Sun, Zheyuan Zhang, Mu Tian, Celine Mougenot, Nick Glozier, Rafael A Calvo

**Affiliations:** 1 Dyson School of Design Engineering Imperial College London London United Kingdom; 2 Luye Medical Group Shanghai China; 3 Faculty of Medicine and Health The University of Sydney Sydney Australia

**Keywords:** mental health, digital health, workplace, China

## Abstract

**Background:**

Workplace mental health is under-studied in China, making it difficult to design effective interventions. To encourage the engagement with interventions, it is crucial to understand employees’ motivation toward seeking help through technologies.

**Objective:**

This study aimed to understanding how Chinese employees view digital mental health support technology and how mental health support technology could be designed to boost the motivation of Chinese employees to use it.

**Methods:**

A mixed methods approach was used. In total, 458 Chinese employees (248/458, 54% female) in 5 industries (manufacturing, software, medical, government, and education) responded to a survey, and 14 employees and 5 managers were interviewed.

**Results:**

Government data and employee responses showed that mental health support in China is limited. In the workplace, Chinese employees experience a lower sense of autonomy satisfaction compared with competence and relatedness. Although managers and employees try to empathize with those who have mental health issues, discrimination and the stigma of mental illness are rife in Chinese workplaces. Digital technologies are perceived as a potential medium for mental health interventions; however, privacy is a major concern.

**Conclusions:**

The results of this study demonstrated the potential of self-help digital mental health support for Chinese employees. Interdisciplinary cooperation between design engineers and mental health researchers can contribute toward understanding the issues that engage or disengage users with digital mental health interventions.

## Introduction

Over the last 2 decades, the link between workplaces and employees’ mental health has become increasingly important to managers, employees, and their families worldwide [1]. In the United Kingdom, 15% of employees experience symptoms of existing mental health conditions [2]. In China, 35% of employees experience difficulties in stress management and 21% to 29% of the working population believe that their mental ill-being has a significant negative impact on their social life and working performance [3].

Despite these dire statistics, few people with mental disorders in China actively seek clinical support, with the rates being 17% in rural areas [4] and 25% in urban areas [5]. This has been attributed to the stigma surrounding mental health, availability of adequate support, and cost of professional interventions. According to Zhang et al [5], 93.6% of the patients and caregivers in China misunderstand mental illnesses, preventing them from receiving appropriate help. There are only 0.036 clinical psychologists per 10,000 Chinese citizens, whereas the figure is 6 in the United States and 1.8 in the United Kingdom [6,7]. As for financial resources, the Chinese government has increased mental health expenditure per capita to US $0.13 by 2018, which is in contrast to the average of US $21.7 in Europe [8]. With the rapid increase in the direct medical costs of mental health, families in China face considerable financial burden, as out-of-pocket payments are the most common payment method for most mental health care expenditures [9].

According to the World Health Organization [10], mental health is defined as “a state of well-being in which an individual realizes his or her own abilities, can cope with the normal stresses of life, can work productively and is able to make a contribution to his or her community.” As a country with rapid transition of economic and social system, the Chinese government is turning its attention to mental health–related issues and their negative impact on productivity [11].

Although programs and research on workplace mental health have grown in the West, occupational mental health has not been widely recognized as a significant research area in China. This has led to a lack of understanding of workplace mental illness, its sources, and the effectiveness of interventions. The understanding of these issues in Western workplaces may not generalize well to Chinese ones, given that the social perceptions of mental illness might be different; for example, patients with mental illness are traditionally considered a possible source of social unrest because of their potentially anomalous behavior [12]. The expectations about the relationship between employees and others from their companies would also differ, as in Chinese society, it is important to maintain *guanxi* (an underlying harmonious social relationship) with each other [13]. To design culturally acceptable digital interventions, a better understanding of the sociotechnical context is essential to meet the preferences and fulfill the needs of the target users. This includes understanding the “local definitions of personhood and the good life” and how they are situated [14].

In the West, digital well-being interventions have shown the potential to reduce the risks of mental illness in the workplace and support those who are already ill with positive acceptance [15]. Meta-analyses from 2017-2021 [16-18] showed that eHealth interventions can reduce the level of mental illness in employees and act as a treatment by reducing illness symptoms in those who are already ill. However, in China, although there are more than 986 million smartphone users [19] as of 2021, there are few studies on workplace digital mental health interventions. Meta-analyses in 2017 and 2019 [16,17] only included 1 study in China—a web-based mental health training program for university staff and students in Hong Kong by Mak et al [20].

In a 2019 study by Peking University Health Science Center [21], researchers conducted a qualitative assessment of all the existing smartphone-based mental health support apps from the iOS market and 3 major Android markets in China. In total, 63 unique apps were identified in the market following the criteria of (1) accessibility, (2) targeting the public (not designed for mental health experts), (3) technical robustness, and (4) evidence-based content. The apps were built on the basis of 2 primary features: 67% of the apps provide mental health education either by informative articles or by relevant courses (14 with free content and 28 with paid content); and 65% of the apps were built to provide videoconference counseling services. Only 7 of the 63 apps contained courses designed on the basis of verified psychological therapy or training. In addition, each app was measured using a Mobile App Rating Scale (MARS) [22] in 4 dimensions: engagement, function, esthetics, and information. Each MARS item uses a 5-point scale (1=inadequate, 2=poor, 3=acceptable, 4=good, and 5=excellent); the maximum scores for the Chinese apps were 4.0, 4.3, 4.0, and 4.0, respectively, much lower than the results of 4.6, 4.75, 4.83, and 4.6 using the same scale on mental well-being apps in the English language [21,22]. This suggests room for improvement in many aspects of the app design.

For the design of effective and acceptable digital mental health support technologies, it is crucial for design engineers to understand “How do Chinese employees view digital mental health support technology, and how could a mental health support technology be designed to boost the motivation of Chinese employees from using it?”

## Methods

### Study Structure Overview

A mixed method user research was conducted to provide insights into Chinese employees’ understanding of mental health and their preferences for interventions, privacy, terms used, platform, and style. This paper discusses 2 studies: a quantitative survey-based study and a qualitative analysis of interviews with employees and managers.

### Study 1: Survey

#### Sample and Recruitment

Employees were invited to the study via internal messaging tools in 5 Chinese local companies and organizations collaborating in the project: a manufacturing company (approximately 500 employees), software company (200 employees), state administration office (1400 officers), medical company (400 employees), and university department (50 students and staff). The factory of the manufacturing company was in a tier-3 city, where mental health resources are more limited [11] than the other 4 companies and organizations in tier-1 provincial capitals.

The anonymous invitation link was published in the companies’ and organizations’ group chat rather than directly sent to individuals to ensure that participation in this web-based survey was voluntary. Note that bias exists, as participants who are familiar with mental health and mental illness were more likely to voluntarily complete the survey; however, in the recruitment messages, participants were encouraged to complete the survey even if they did not have previous experiences with mental well-being support. The link to the survey was open for 1 month.

Instead of emails, messaging tools such as “WeChat” and “DingTalk” are more frequently used for the communication between colleagues in China. To disseminate the survey, we chose to send messages in the company’s group chat. The exact number of employees who read the messages in the group chat was unknown.

To be eligible, participants had to be Chinese residents, older than 18 years, and working in one of the 5 companies or organizations. Participants were provided with an information sheet describing the study and requested to sign a consent form before the start of the survey.

From the recruitment of participants to the end of the questionnaire collection, none of the cities, where the companies and organizations were based, were in COVID lockdown.

#### Survey Structure

The first study was conducted in the form of a web-based survey in simplified Chinese with WJX (“问卷星,” a Chinese web-based survey platform). The survey took 10-15 minutes to complete, and its questions were organized into the following sections using standardized questionnaires when available:

1. Demographic information (gender, age, and role in the company) was measured using generic options provided by WJX.

2. History of mental health issue (“Are you currently or previously undergoing any form of psychological treatment?”)

3. Smartphone use: internet use, current use, and satisfaction with apps for stress relief

4. Work motivation: psychological needs satisfaction (Basic Psychological Needs at Work Scale [BPNWS]) [23]

5. Mental well-being supportive preference: experience of and hindrance to receiving mental health support, experience of using and willingness to use different types of digital mental health support, preferred features for digital mental health support (based on the list of features recorded in a previous study for the Headgear app) [24]

6. Further participation (if the participant wanted to be contacted for the interview, they could leave their contact details).

#### Data Analysis

Statistical results collected from the backend database of WJX were first analyzed by industry and then aggregated to make an overall sample of the working population in China.

According to the self-determination theory [25], the satisfaction of the 3 basic psychological needs of autonomy, competence, and relatedness is positively related to psychological well-being and workplace engagement and negatively related to ill-being. In this part of the survey, the participants were invited to complete the BPNWS [23], which is a 12-item instrument on a 6-point scale. In a study by Brien et al [23], the French and English versions of the BPNWS were validated. The scale was translated into Chinese by 2 bilingual researchers. The Cronbach α coefficient was used to validate the translated version of the scale. One-way ANOVA was used to investigate the differences in basic psychological needs among the 5 industries.

### Study 2: Interview

#### Recruitment

**Figure 1:**
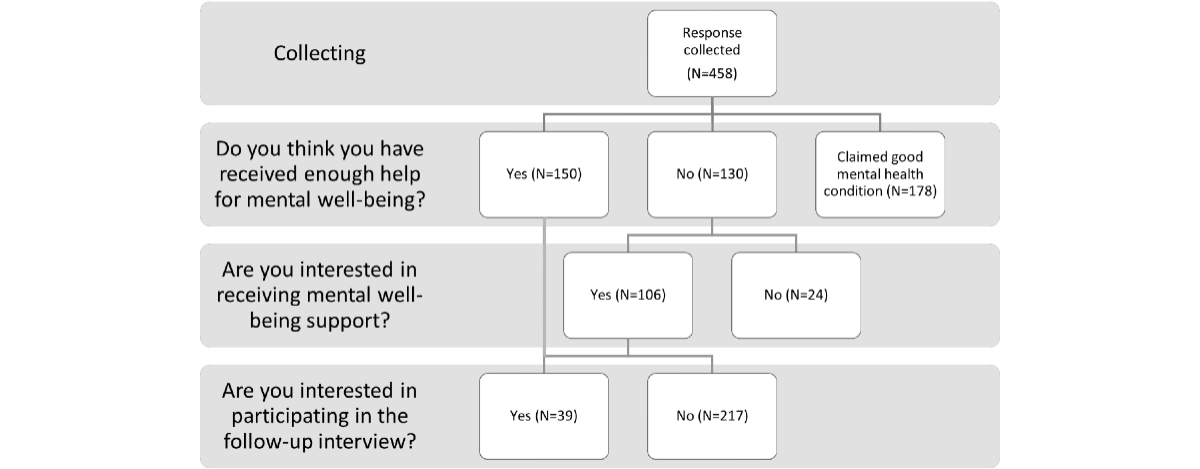
Interview recruitment logic.

Participants in study 1 who expressed an interest in mobile-based mental health support technology and left their personal contact details for the follow-up interview, were invited to study 2. The logic of the selection is demonstrated in Figure 1.

A total of 39 eligible participants voluntarily left their contact details for the interviews in study 2. The final study size was 14, as interviewees started to discuss recurrent themes.

In addition, 5 managers from the same companies and organizations were invited to participate in the interviews.

#### Interview Structure

In the study 2, detailed questions about perspectives on mental well-being were asked in the form of semistructured interviews held in Mandarin. The interview structure was designed to inspire the participants to openly share their opinions and ideas about digital support for workplace mental health.

Each interview took approximately 30 minutes, covering the following topics:

1. Experience of workplace stress and stigma toward mental health problems
2. Current way of dealing with workplace stress
3. Perspectives on mobile mental health support technologies in general
4. Feature requirements for a mobile mental health support technology
5. Other suggestions on mobile mental health support technology (art style, platform, privacy, etc)

Separate interviews were carried out with managers of each organization, discussing the current mental health support provided by the organization and their opinions on the mental health problems of employees in the workplace.

#### Data Analysis

Qualitative text data were manually transcribed from interviews by a Mandarin-speaking native Chinese researcher. Inductive thematic analysis was conducted on the transcribed text by 2 bilingual researchers (both had received university-level education in the United Kingdom) using a qualitative data analysis software, NVivo (QSR International). This study aimed to understand what motivates employees to use digital mental health support; therefore, the results were categorized into the following 3 main topics: their current ways of handling their stress, their opinions toward mental health in the workplace, and their ideas for digital mental health support technology. The text quotes were initially identified and differentiated into the above categories in the first round of coding and then iteratively subjected to inductive thematic analysis to develop 5 sets of views on stress, relaxation, workplace support, workplace mental health, and digital support technology. The last set of views about the technology consists of their requirements and aversions to features, media, structure, privacy, and esthetics.

### Patient and Public Involvement

In this study, we did not inquire about diagnosis or treatment and did not talk about “patients” but rather about end users or the “public.” They were welcomed to discuss any interests and concerns regarding digital mental health support technologies in the semistructured interviews. End users were not involved in the study design.

### Ethics Approval

This research was approved by the Imperial College Research Ethics Committee (ethics code 21IC6579).

## Results

### Survey Results

#### Demographics

**Figure 2:**
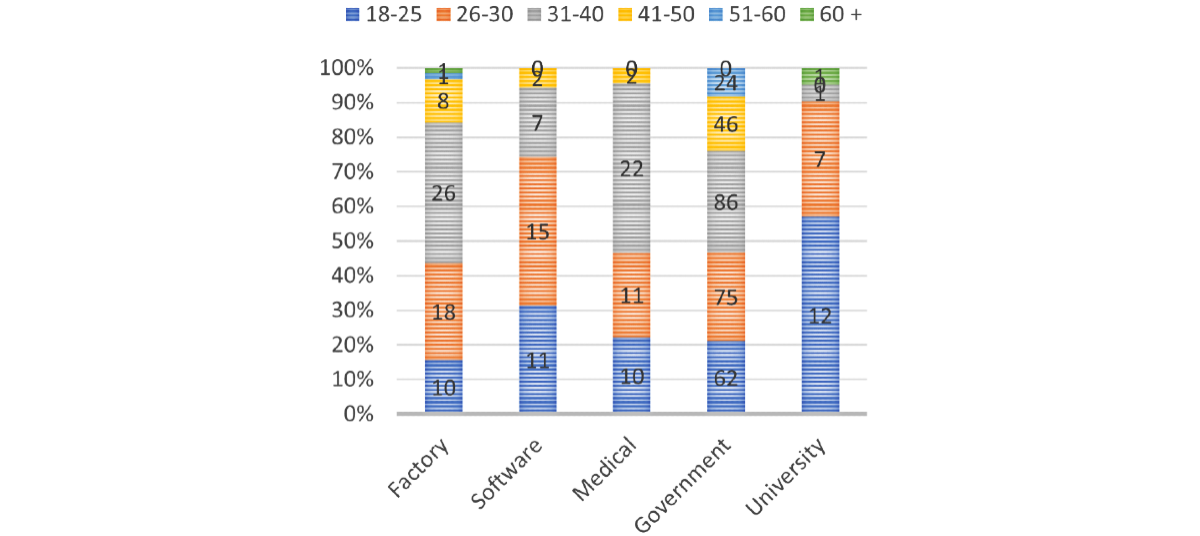
Age distribution (in years).

**Figure 3:**
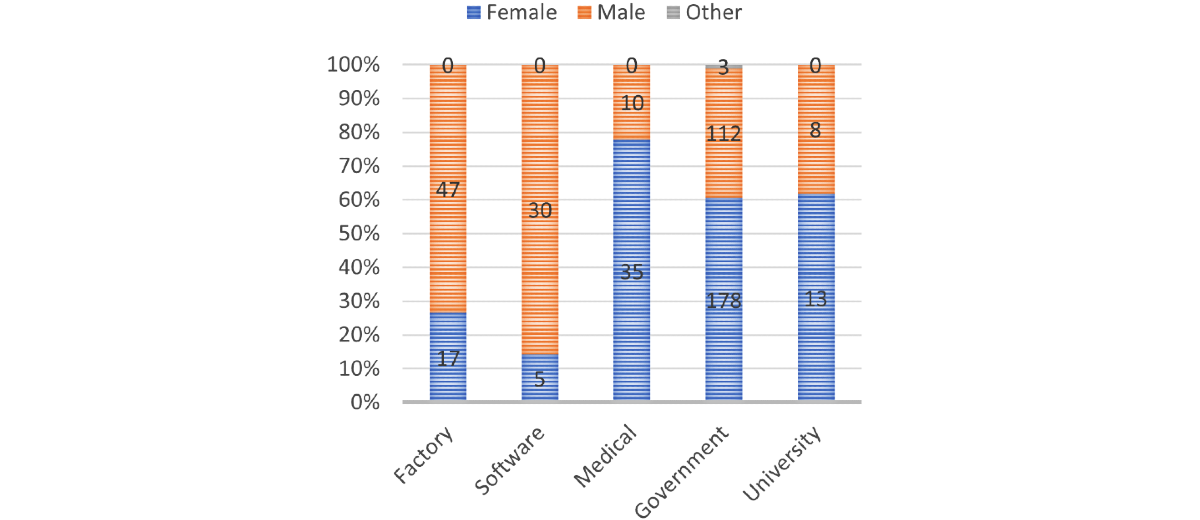
Gender distribution.

A total of 458 participants completed the survey between June 2021 and November 2021 with a mean age of 32.7 (SD 9.5) years, ranging from 18-65 years (248/458, 54.3% female and 207/458, 45.3% male, as shown in Figures 2 and 3). Of the 458 participants, 14% (n=64) of the participants came from a manufacturing factory (RR [response rate]≈12.8%), 7.6% (n=35) of the participants from a software company (RR≈17.5%), 9.8% (n=45) of the participants from a private medical group (RR≈11.3%), 64% (n=293) of the participants from the government administration (RR≈20.9%), and 4.6% (n=21) from a university (8 students and others were teachers or administrative staff). Of all the participants, 2.4% (11/458) of the participants were undergoing clinical treatment for mental health problems (in any form, including medication and psychotherapy), and another 4.1% (19/458) of the participants had been treated before, with anxiety (18/30, 60%) and depression (16/30, 53%) being the 2 main conditions.

Of the 64 employees in the manufacturing company, which was located in a tier-3 city, 30 (47%) of employees were blue-collar production workers and 3 (5%) participants were either under treatment or had been previously treated. In tier-1 cities, this rate was higher (27/394, 6.85%), probably owing to the wider availability of mental health support resources.

#### Smartphone Use

Understanding how the target population uses mobile phone technology provides valuable design insights. Of the 458 total participants, 457 (99.8%) participants reported smartphone use, with a mean use of 3.6 (SD 3.06) hours per day. Bandwidth is potentially an issue for 15% (69/457) of users; 63% (288/457) of the smartphone users have monthly data plans; and another 22.3% (102/457) have unlimited data plans. Most already see their phones as devices that can contribute to well-being; 73.5% (336/457) of smartphone owners use them for relaxation in their daily lives, with video and streaming apps being the top choice for mobile-based relaxation (209/457, 45.7%) followed by music apps (164/457, 35.9%), shopping apps (151/457, 33%), news and article apps (135/457, 29.5%), social media apps (133/457, 29.1%), and casual games (124/457, 27.1%). However, only 5 (1.1%) of the 458 participants used dedicated mental health support apps for relaxation.

Smartphone users were asked whether they would consider using a phone app to help them optimize their mental well-being. Furthermore, 50.5% (231/457) of the participants claimed that they were not interested in any form of support, as they were either satisfied with their current mental health status or had received sufficient support.

Additionally, 49.5% (226/457) of the participants demanded mental health support, 25.8% (118/457) would consider using an app, and another 21%(96/457) would consider taking help if it was in the form of a WeChat mini-program (a popular lightweight microapp embedded into the social messaging platform of WeChat) instead of a stand-alone app. Only 2.6% (12/457) of the participants responded negatively to help from digital technology. Of the 12 participants who resisted digital support, 10 (83%) of them had not been diagnosed with mental health difficulties and had not received any diagnosis of any kind. The other 2 (17%) employees who were receiving treatment preferred face-to-face clinical support from experts.

#### Current Mental Health Support

The participants’ attitudes toward mental health support were investigated. Of the 458 participants, 38.9% (n=178) of the participants believed that they did not need any form of support, and another 32.8% (n=150) believed they had received sufficient help (note that this result is not the same as in section 3.1.2, as participants who are satisfied with their current support may still be interested in testing out such an app). For the 130 (28.4%) out of 458 participants who did not receive enough help, “I do not have time” (62/130, 47.7%), “I do not know where or how to seek help” (59/130, 45.4%), and “I do not want to worry friends and family” (54/130, 41.5%) were the top 3 concerns. Of the 130 participants, 106 (81.5%) wanted further mental health support.

Self-help (166/257, 64.6%) was the most desired way of support, followed by peer support (153/257, 59.5%) and face-to-face expert support (107/257, 41.6%). The preference for self-care is in line with acceptance of mobile-based mental health support.

#### Basic Psychological Needs at Workplace

According to the self-determination theory [25], the satisfaction of the 3 basic psychological needs of autonomy, competence, and relatedness has a positive relationship with psychological well-being and workplace engagement and is negatively related to ill-being. In this part of the survey, the participants were invited to participate in the BPNWS [23], which is a 12-item instrument on a 6-point scale. In the study by Brien at al [23], the French and English versions of the BPNWS were validated. The scale was translated into Chinese by 2 bilingual researchers. The results suggested that the reliability of the Chinese version of the scale was above good, with Cronbach α coefficients of 0.82, 0.92, and 0.93 for autonomous, competence, and relatedness, respectively. Compared with the French and Canadian teachers who were assessed using BPNWS by Brien et al [23], Chinese employees in general showed lower satisfaction scores for autonomy (mean 4.21, SD 1.40) but similar results for competence (mean 4.88, SD 1.06) and relatedness (mean 4.49, SD 1.16).

We note that the interpretation of the results is limited, given that the study was on teachers, whereas our study had participants from several industries.

ANOVA was conducted across the 5 industries separately for autonomy, competence, and relatedness. The mean scores for satisfaction with the 3 basic psychological needs are listed in (Table 1). No significant differences were observed in competence (*P*=.75) and relatedness (*P*=.25), but significant associations were shown in the mean scores for autonomy (*P*=.005) by industry. Post hoc tests suggested that the mean autonomy score of employees from the private medical company and university was significantly higher than the mean scores of employees from the government (*P*=.02 [medical company vs government] and *P*=.002 [university vs government]) and the factory (*P*=.01 [medical company vs factory] and *P*=.005 [university vs factory]). This is in line with the results of section 3.2.2, where in the interview, boredom was repeatedly mentioned by the government officers as a workplace stress source. Research in medical companies and universities could give employees more freedom to control their own work.

In addition to understanding what might drive mental ill health, it is useful to understand what drives the individuals to seek help.

**Table 1:** Mean scores (and SD) of basic psychological needs satisfaction for the 5 industries.

	Software company	Medical company	University department	Government office	Factory
Autonomy, mean (SD)	4.29 (1.02)	4.64 (0.81)	4.68 (0.69)	4.10 (1.13)	4.18 (0.82)
Competence, mean (SD)	4.81 (1.15)	5.02 (0.61)	4.93 (0.60)	4.87 (0.98)	4.89 (0.86)
Relatedness, mean (SD)	4.49 (1.25)	4.54 (0.86)	4.48 (0.68)	4.54 (1.08)	4.21 (1.07)

#### Feature Preferences

The 214 participants who showed an interest in the smartphone-based mental health app were asked to select their desired features. There were no limitations to the number of features selected. The top 10 feature preferences are summarized in Table 2, showing the following priorities.

**Table 2:** Feature preferences (N=214).

Rank	Features	Participants, n (%)
1	Mood-fix tool	127 (59.3)
2	Talk with experts	121 (56.5)
3	Book and article recommendation	117 (54.7)
4	Self-assessment quiz with feedback	109 (50.9)
5	Ability to record the mood triggers	109 (50.9)
6	Dashboard showing progress	108 (50.5)
7	Emotion tracking	101 (47.2)
8	Workouts and exercises	100 (46.7)
9	Suggestions based on self-assessments	98 (45.8)
10	Relaxing music	98 (45.8)

### Interview Results

#### Stress Source

Participants reported 9 main sources of workplace-related stress. These stress sources are classified into 2 broad categories based on the Job Demands-Resources model [26]. An imbalance between demands on employees and the resources they possess to deal with those demands leads to workplace stress.

Job demands including dealing with unreasonable clients, meeting deadlines, giving a public speech, reaching target key performance indicators, and taking on responsibilities are causing job strain. The lack of job resources had a further negative impact, including boredom, managerial difficulties, insufficient support from teammates, and low confidence in obtaining promotions. Both high job demands and limited job resources contributed to stressful working experiences.

In addition to work-related issues, interviewees expanded the topic to express their concerns for other stress sources, including family, finance, commuting, and health conditions. For future development of workplace mental health support, we need to bear in mind that we should not only solely focus on work-related problems but also pay equal attention to other factors.

#### Methods to Relieve Stress

Talking about ways to relieve stress, participants held a common ground that “To get to the root of the stress problem, the best solution is to address the issue as quickly as possible.” However, if it could not be solved in a timely manner or required support from others like experts, it was common for the employees to try to address it by talking to their friends and colleagues, practicing outdoor activities, or relying on digital devices for relaxation (see section 3.1.2).

Employees also mentioned that their companies or organizations provided human resource consulting, team building activities, mental health training lessons, relaxation rooms, gyms, and massage sofas as mental health support.

#### Attitudes Toward Mental Health

Most employees (who were coded as [E1] to [E14] to label their quotes) acknowledged the importance of mental health education and support when discussing their opinions on mental health:

It is common for people to feel depressed or anxious at times. Everyone has a certain threshold for taking up stress. When that threshold is reached, he will lose his control for a while.

E5

Most interviewees showed understanding and sympathy toward people with mental health difficulties. They could recognize depression or anxiety as real medical illnesses and would actively encourage those experiencing these problems to speak out to them or seek professional support.

However, when they evaluated people with mental health difficulties from the perspective of working partners, interviewees would lower their expectations of their colleagues’ abilities in many aspects including communication, teamwork, capability, problem solving, time management, and work motivation.

Interviewees showed a tendency to maintain a distance from mentally unwell people for their own safety and that of the person:

People who are affected by negative emotions are prone to anger and temper tantrums.

E2

If I knew this person had anxiety, I would definitely be wary of this person. Because I would have concerns about that if he suddenly became irritable.

E7

Stigma was another topic that was repeatedly discussed in the interviews. Interviewees described most people as holding “conservative” views on mental health, as discrimination toward mental illness is common in the Chinese culture. In Chinese, the word “mentally ill” (“精神病”) is widely used as a cursing word to criticize the insanity behind someone’s misbehavior. Advising someone to go see a psychologist is considered “extremely impolite and unacceptable.” Thus, employees with mental difficulties will make their best efforts to hide their conditions to avoid underestimation of working abilities by their colleagues or leaders. However, based on their own experiences and examples around them, most (10/14, 71.4%) interviewees admitted that covering it up was merely a means of protecting the sufferer from further social pressure, which might lead to a “vicious circle” of stress accumulation. This creates the requirement for self-help technology for mental health support.

#### Company Organization Support

Government administration interviewees claimed that they were not provided with mental health support services. However, there was a “relax room” in every office site where employees could rest, sit on the massage sofa for a while, and play table tennis. In addition, once a year, a lecturer might be invited to talk to staff about mental training. However, staff found it less helpful because of the few training programs.

Employees in manufacturing and software companies referred to talking with human resource managers and team building as mental well-being support provided by the company.

Managers in these companies claimed that the reasons they did not set up professional mental health support included the cost to the company and the privacy concerns of employees that would hinder their engagement. It was also difficult for them to assess the benefits of investing resources into mental health support.

#### Features Requirements

From the qualitative analysis of the 14 interviews, 8 topics were repeatedly identified for digital mental health support. These features include the following:

1. Self-assessment
2. Education
3. Mood-fix tools
4. Self-help exercise
5. Seek help from experts or community
6. Design style
7. Worries
8. Trust

We will elaborate more on each topic.

##### Self-assessment

The first common expectation of employees was to judge their current state of mental health:

I would like the app to give me an understanding of my mental wellbeing conditions, preferably through a structured medically supported diagnosis. There should be questions to determine what level of problem you are at, thus suggest how to relax, whether you need to go to hospital, or even, whether you need to be hospitalised.

E1

Because I don’t have psychological knowledge, I don’t know if I have a problem. I would like to judge it for myself with the help of theoretical instructions.

E4

##### Education

Employees showed interests in learning trustworthy psychological knowledges:

I am looking for books and articles on the subject. I want to logically sort out where my emotions come from.

E11

It is like getting a cold, once a man sneezes, his common senses tell him that he is having a cold. One should learn some common senses while he is still healthy. Thus, once he does get into unpleasant situation, he knows he may be depressed or not in a normal state of mind.

E5

Participants also expected that with the help of the self-assessment feature, it would be most helpful to recommend personalized psychoeducation contents to users.

In addition, employees were keen to acquire workplace-specific knowledge as part of their mental self-help. This included how to overcome workplace crises, how to communicate with leaders and colleagues, how to deal with workplace bullying, and how to handle stress through time management:

When you encounter a crisis at work, you may need some tips from psychological perspectives. There are so many contradicting articles on the internet, but I don’t know what is right and what is wrong, so I hope I can get access to some articles or content that is really valuable.

E11

It will be interesting to learn about how other countries have dealt with workplace mental health. Because China is at a very early stage in this area, people may not have any idea, so it helps to give some examples of how foreign countries with a more developed mental health support system deal with these problems.

E6

Short videos are the most welcomed media for receiving psychoeducation, and many participants mentioned TikTok as their favorite way of relaxation using digital devices.

##### Mood-Fix Tools

Participants expressed a strong demand for tools that could help them quickly fix their mood during a workplace mental crisis that leads to moments of depression or anxiety.

Based on their current methods of quick mood-fixes using digital devices, they suggested soothing music, humorous short stories, casual games, and breathing exercises.

##### Self-help Exercises

Employees demanded mental health supportive exercises, both spiritual and physical.

Although meditation is less known as an evidence-based practice for mental health support, its connection to Buddhism draws interest, as Buddhism plays a vital role in the spiritual aspects of Chinese culture:

There are some useful practices in Buddhism, such as meditation, selflessness, letting go of obsessions, yoga. It’s about de-emphasising self-consciousness and relationships with people, making yourself pure and calm. Relieving your stress by thinking of problems in a more objective perspective.

E6

I want to learn meditation through a tutorial with daily exercises.

E4

However, some interviewees raised concerns over conditions for meditation:

I think meditation is very useful, but it requires willpower and a quiet and relaxing environment, which many people don’t have.

E11

Physical activities including exercises and sleep quality improvement exercises are also demanded:

Add some exercises, such as simple stretches or office exercises where you can practice in a chair.

E6

Sometimes when we go to bed and wake up, many of our worries are gone, but if we don’t get a good night’s sleep, even if we don’t have any worries, they will arise. The endocrine system is also affected. It would be good if there was an app that could help with sleep.

E12

##### Seek Help From Experts or Community

Some employees expressed a desire to communicate with experts or peers. However, others had different attitudes toward price, efficacy, and privacy:

If they can’t solve it by self-help, the app can recommend some psychologists or other clinical treatments.

E13

I think that, for social purposes, it might be more effective to find people on the Internet who share the same goals and talk about them. But there are ethical issues involved.

E4

Responsible people may be reluctant to talk to their families about the pressures at work. Some incognito channels of communication with the leader can be helpful.

E10

I often read psychological articles in Zhihu (A Chinese question-and-answer website). Sometimes I don’t like things that are particularly official. It’s interesting to see the answers to a certain question from various perspectives. People want to find others who can understand them. However, in a community like this, where you ask a question and others will answer and discuss it, sometimes it can distort your perceptions and make the problem even worse. Sometimes, you still need to take guidance from professionals.

E11

I think that when it comes to approaching a psychiatrist, the first concern is probably the price. You will be worried what if, in fact, these people are merely there to give you ambiguous advice for money.

E6

##### Design Styles

Participants actively described their preferences for digital support in the study, and their ideas were summarized using the following 3 keywords as shown in Textbox 1.

**Keywords and ideas.**

**Mind-opening**

- *“contents that exposes me to new stuff and holds my attention”* [E3]

**Straightforward**

- *“The only thing I am willing to do when I am stressful is to do simple relaxing activities following instructions from others.”* [E12]
- Rather than making the users think overly complicated, the participants preferred plain and straightforward wording and exercises.

**Flexible**

- *“I tend to have more freedom. Don’t want to stick to all those rules and regulations. I prefer a bit of simplicity.”* [E14]

##### Worries

Of the 14 participants, 5 (36%) questioned the possible effectiveness of using a mental health support technology. For example:

Being stressful is because the task is not finished and there are many difficulties in finishing it. If you let me play games or listen to music for a short time, because I still have unfinished business in my mind, in the long term it won’t help and may put on further stress.

E2

When I feel stressful, temporarily letting go of my anxiety could make me even more stressful afterwards.

E11

Employees were also concerned about receiving contents that they found inappropriate, especially tedious sermons and advertisements:

I hate being lectured, ‘You should do this,’ ‘You shouldn’t do that.’ I’d rather have no one around than someone there to lecture me.

E10

When you are stressed, you don’t want to study, you don’t want to get overwhelmed by lecturing texts, and you certainly don’t want to see any advertisements.”

E13

I don’t want to see any advertisement. I will doubt if the contents are really valid or are they just trying to lure me to pay for their services.

E3

##### Trust

At the end of the study, participants were asked about their trust in provider of mental health support technology.

All the interviewees showed a lack of trust in their companies, fearing that their privacy would be violated by exposing their mental health conditions to their employers. In contrast, people place more trust in government and nonprofit organizations.

#### Managers’ Perspectives

In addition to the interviews with employees, 5 managers (1 from the government office, 3 from the factory, and 1 from the software company) were invited for interviews before starting the survey. They were either human resource directors or executives of the companies or organizations. Team key performance indicators and team management were the primary sources of stress for managers in the workplace.

From a personal perspective, most managers claimed that employees with mental health problems were understandable and should not be discriminated against. However, from the standpoint of the company or organization, they were not likely to hire those with mental health issues. This sentiment is reflected in a quote from a manager of a manufacturing company:

Workers live and eat together so that it will have a bad influence.

Most of the time, I will not hire someone with mental health or anxiety problems. Unless his or her mental health condition somehow suits his position. For example, if his or her anxiety is caused by perfectionism, he or she may be a suitable candidate for quality inspection tasks.

I will not hire someone with mental health problems. Especially for salespeople. I don’t believe they have the ability to communicate well with the customers.

When managers were asked about the measures they would consider taking if one of the employees was found to experience mental problems, they held different opinions:

Employees with anxiety problems are acceptable if they do not negatively affect others. A negative employee may have a bad influence on the work atmosphere, and thus make others lose confidence in dealing with the current problem and hard to collaborate.

Anxiety may be caused by incompetence. However, most of the time, the problem is that he or she is put into the wrong position. Changing his type of work may help.

I will encourage them to stop working and seek interventions. Employees with severe mental health problems may pose physical threats to their colleagues.

If we found some employee currently suffering from anxiety problems, we will advise him to quit the job.

The managers mentioned that their expectations for employees with mental problems would be lowered, especially in their work performance and time management ability.

All the managers believed that a mental health support app would benefit both the employees and the company. However, cost is the top concern that prevents companies from setting up mental health services for employees. Moreover, even if managers are willing to provide the services, they do not know where to start. This is because of the lack of professional training and guidance in relevant fields for managers.

## Discussion

### Principal Findings

The results of study 1 showed that 46.3% (106/226) of the participants who were willing to receive mental health support felt that they had not received enough help. Participants in study 2 also reported a limited provision of mental health support services by the companies. This finding is in line with previous studies (as described in section 1), which reiterates that stigma and lack of resources deter people in China from seeking mental health support.

The results from section 3.1.4 suggest that Chinese employees may feel less autonomous than their French and Canadian peers, with mean satisfaction scores lower than those of both Western countries. However, this result requires further validation, as the satisfaction of autonomy needs differs across industries in China. The evidence suggests that support could be developed to prevent stress by guiding employees to satisfy their sense of autonomy, as the lack of autonomy can lead to workplace stress according to the theory of Job Demands-Resources model [26].

Although the participants in study 2 showed empathy and understanding toward people with mental health conditions, discrimination still widely exists. People with mental health conditions are criticized for their weaknesses and tend to underestimate the various abilities required by their jobs. Employees are usually rejected or even dismissed if their mental health conditions are exposed to the company. Thus, they tend to cover their true emotions, leaving them less likely to receive appropriate help and support. This self-concealing of emotions and feelings together with the Asian culture of “not wanting to worry friends and family*”* makes self-help (166/257, 64.6%) the most desired way of digital mental health support.

As employees spend a lot of time on their smartphones with internet access, it is common to rely on them for relaxation and mental health support. Although very few of them benefited from dedicated mental health support apps, most were willing to try out such a technology, provided it was effective.

As explained in section 3.1.5, study 1 showed employees’ requirements for features in the 5 categories: self-assessment, self-guided support, psychoeducation, emotion tracking, and expert counseling.

**Figure 4:**
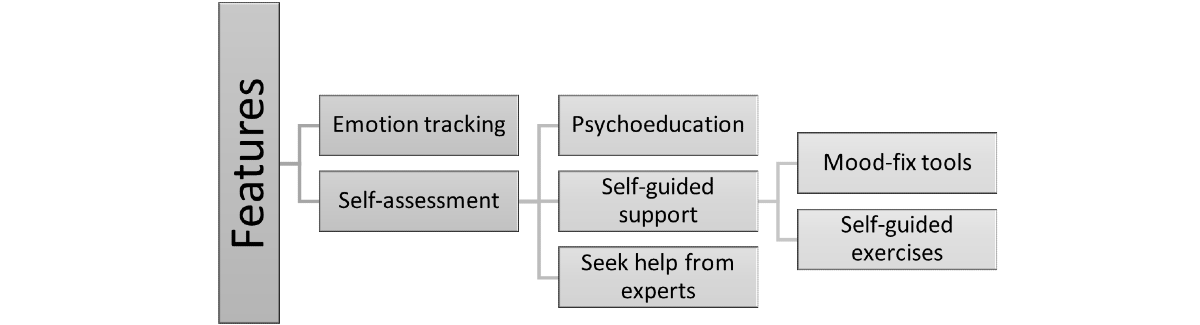
Digital support features requirements.

Combined with the results of study 2, the features are summarized in the logic shown in (Figure 4). In addition to the feature of emotion tracking and corresponding triggers, employees would like to take self-assessments to understand their current mental health conditions and based on this receive psychoeducational material, access to self-guided support, and seek help from experts accordingly.

The statistics also favored the deployment of digital mental health support on a WeChat “mini-program,” as users can access the contents in the mini-programs immediately without downloading, installing, or signing up.

As revealing one’s mental health problems can lead to underestimation and alienation, people take their privacy seriously. As discussed in section 3.2.5, employees are more likely to trust and adopt support from publishers such as the government or nonprofit organizations that are not related to their employment. As a part of the “National Mental Health Working Plan (2015-2020),” the Chinese government is actively experimenting community-based support to promote mental health [11]. With central government endorsement, introducing self-help mental health support by the community is more likely to result in increased motivation than that by the company. This could impact the development of Employment Assistance Program services in China. The providers need to convince employees that their information and records will not be accessible to their employers, gain their trust, and promote their motivation to use the service.

### Limitations

The main limitation of this study is the potential bias in the sample participants. Although the participants were selected from 5 companies or organizations with diverse backgrounds, all these companies and organizations are located in large cities with more than 4 million residents. Except for 6.6% (30/458) of the factory workers, all other employees in the study could be classified as white-collar workers living in urban areas. The mental health condition and support preferences of workers living in less developed cities and rural areas could be quite different from these results [4,5].

The limitation of the survey dissemination method is that if the employee was not on the web portal during the spread of the survey link, the group chat was likely to be flooded by other messages, so the employee might not have the chance to read the invitation at all. Therefore, it was impossible to calculate the number of employees who read the message, resulting in a much lower RR.

In addition, the results are likely to have been influenced by self-selection bias, especially in study 2 where voluntary participants are likely to represent individuals who are more open to mental health issues.

### Future Work

Design engineers, working in collaboration with mental health researchers, can contribute to understanding the issues that engage or disengage users with digital mental health interventions. In the West, such collaborations are increasingly common as shown by the emergence of journals and workshops such as [27,28]. In China, the adoption of such design procedures is still in the exploration stage. We aim to bridge this gap by designing, developing, and piloting further research.

### Conclusions

Our study across 5 companies and organizations in China showed that the proportion (150/458, 32.8%) of employees in these companies and organizations who were accessing any form of mental health support was very low, both from clinical treatment and digital support via smartphones. However, more than a quarter (118/457, 25.8%) of the respondents reported that they liked much more smartphone-based mental health support, indicating an exceptionally large need and demand for such services. The qualitative interviews demonstrated problems caused by stigma around mental ill-being, such as unwillingness to disclose and low help-seeking rates, either in clinical services or within the company or organization. This suggests that the way forward is to develop confidential and anonymized apps that enable people to undertake self-help to improve their mental health. Certain elements of features were deemed very desirable, including tools to help people fix their moods and professional guidance either in person or with related books and articles. This will help us to think about how to develop the content of a self-help app to enable Chinese employees to improve their mental health not only focusing on work stress but also on other stressors in their lives.

## Abbreviations

BPNWS: Basic Psychological Needs at Work Scale
MARS: Mobile App Rating Scale
RR: response rate
